# Association between peripheral blood/bronchoalveolar lavage eosinophilia and significant oxygen requirements in patients with acute eosinophilic pneumonia

**DOI:** 10.1186/s12890-020-1056-7

**Published:** 2020-01-28

**Authors:** Joon Young Choi, Jeong Uk Lim, Ho Jung Jeong, Ji Eun Lee, Chin Kook Rhee

**Affiliations:** 1 Division of Pulmonary, Allergy and Critical Care Medicine, Department of Internal Medicine, St. Vincent’s Hospital, College of Medicine, The Catholic University of Korea, Suwon, Republic of Korea; 2 Division of Pulmonary, Allergy and Critical Care Medicine, Department of Internal Medicine, Seoul St. Mary’s Hospital, College of Medicine, The Catholic University of Korea, Seoul, Republic of Korea; 3 Division of Pulmonary and Critical Care Medicine, Department of Medicine, The Armed Forces Capital Hospital, Seongnam, Republic of Korea, 222, Banpo-daero Seocho-gu, Seoul, 06591 Republic of Korea

**Keywords:** Acute eosinophilic pneumonia, Eosinophilia, Retrospective study

## Abstract

**Background:**

We investigated the association between a combination of two markers, peripheral (PEC) and bronchoalveolar lavage (BAL) eosinophil percentage (BEP), and oxygen requirements in patients with acute eosinophilic pneumonia (AEP).

**Methods:**

We retrospectively reviewed the medical records of patients with AEP treated at the Armed Forces Capital Hospital between May 2012 and May 2017. We used correlation analyses to assess the association between PEC/BEP and clinical outcomes in AEP patients. Receiver operating characteristic (ROC) curve analyses were used to calculate the cut-off value for BEP that categorised patients requiring a significant oxygen supply. The BAL/blood eosinophil (BBE) score was introduced to stratify patients with peripheral eosinophilia and elevated BEP. Clinical characteristics and outcomes were compared between the different groups. Multiple logistic regression was performed for significant oxygen requirements using two different models using age, C-reactive protein (CRP), smoking duration, and BBE score (model 1) and age, CRP, BEP, and PEC (model 2).

**Results:**

Among the 338 patients, 99.7% were male, and their mean age was 20.4 ± 1.4 years. Only 0.6% of patients were never smokers and the mean number of smoking days was 26.2 ± 25.4. Correlation analyses revealed that both the PaO_2_/FiO_2_ ratio and duration of oxygen supply were associated with BEP. ROC curve analyses indicated a cut-off level of 41.5%. Patients with a high BBE score had favourable outcomes in terms of hypoxemia, hospital days, intensive care unit admission, oxygen supply days, and steroid treatment days. Multiple logistic regression revealed that BEP and BBE score tended to be associated with significant oxygen requirements.

**Conclusions:**

In this study, we revealed that both peripheral and BAL eosinophilia is associated with favourable outcomes in AEP patients.

## Background

Acute eosinophilic pneumonia (AEP) is an infrequent inflammatory lung disease of unknown aetiology [1, 2], which is accompanied by acute respiratory symptoms and diffuse pulmonary infiltration by eosinophils, among other issues [3–5]. The initial clinical presentation and clinical course of AEP can differ from a mild respiratory disease that resolves spontaneously to acute respiratory distress syndrome [6, 7].

In previous studies from our centre, the Army Forces Capital Hospital (AFCH), a military hospital in Korea in which more than 70 patients with AEP are diagnosed annually [8–10], an association between elevated peripheral eosinophil count (PEC) and milder disease severity in AEP was demonstrated [11, 12]. Patients with initial peripheral eosinophilia had significantly higher oxygen saturation and a shorter duration of oxygen treatment compared to patients without peripheral eosinophilia [12].

In addition to peripheral eosinophilia, the correlation between eosinophilia in bronchoalveolar lavage (BAL) fluid and clinical outcomes of AEP has also been evaluated. Previous studies have shown that patients with higher average BAL eosinophil counts (BEPs) have a higher arterial oxygen tension (PaO_2_)/inspiratory oxygen fraction (FiO_2_) ratio [5, 12]. Furthermore, hypoxemia is less severe in patients with higher BEP [13]. Although separate associations between peripheral blood eosinophil counts (PECs) and BEPs and AEP severity have been demonstrated in previous studies, a combination of these two markers may have superior predictive value for clinical outcomes for such patients.

Hence, in this study, we evaluated the clinical characteristics and outcomes of patients who were diagnosed with AEP at the AFHC during the last 5 years.

## Methods

### Study patients

All consecutive patients diagnosed with AEP at the AFCH (tertiary referral military hospital with 874 beds in South Korea) between May 2012 and May 2017 were retrospectively reviewed.

The AFCH is the highest ranking Korean military hospital where all military personnel in need of bronchoscopy are transferred from smaller hospitals including The Armed Forces Busan Hospital, The Armed Forces Cheongpyeong Hospital, and 14 other military hospitals.

Medical records from the electronic medical records (EMR) system and picture archiving and communication system (PACS) were reviewed to confirm AEP cases. Diagnoses were made according to previously proposed criteria as follows: acute-onset febrile respiratory manifestations < 1 month in duration, bilateral diffuse infiltrates observed in chest radiographs, > 25% eosinophils in BAL fluid or eosinophilic pneumonia according to a lung biopsy, and absence of a known cause of pulmonary eosinophilia, including infections, toxins, and drugs [5]. We also included patients who did not undergo BAL or a lung biopsy, and diagnosed AEP by clinical impressions, radiological findings, and peripheral blood test results [10].

### Data collection

We retrospectively collected data from the EMR including general baseline characteristics, detailed smoking history including smoking duration, hospital days, intensive care unit (ICU) admission days, oxygen requirements, initial treatments, and laboratory data including white blood cell (WBC) counts, eosinophil differentials (both blood and BAL fluid), C-reactive protein (CRP) levels, and PaO_2_/FiO_2_ ratio. We also reviewed the PACS to identify chest radiographic findings, including pulmonary infiltrates and pleural effusion.

### Study endpoints

The primary endpoint of our study was total days of oxygen supply during admission, and we further determined which baseline parameters were associated with significant oxygen requirements (total days of oxygen supply ≥2 days after admission) and also evaluated if blood eosinophil (%) and peripheral eosinophil count show significant correlation with total days of oxygen supply.

### Definition of cigarette smoking duration

Considering the unique background of patients included in our cohort, we only counted recent smoking experience. All enrolled patients were military personnel who must undergo basic military training upon entrance to the military. Smoking is strictly prohibited during basic military training of at least 6 weeks. We only evaluated smoking experience after basic military training regardless of past smoking experience.

### Oxygen supply

Oxygen was prescribed to achieve a target saturation of 94–98% [14]. If the patients’ oxygen saturation was not maintained at the optimal level (94–100%) despite oxygen supply by a nasal cannula or mask, oxygen was supplied by a high-flow nasal cannula (HFNC) [15].

Due to a risk of hypoxemia while obtaining BAL fluid, supplemental oxygen was given to the patients during bronchoscopy [16]. To exclude the possibility of including procedural- related oxygen supplementation, which we did not consider significant if the total duration of oxygen supply was only 1 day, and we defined “significant oxygen requirement” as total days of oxygen supply ≥2 days after admission.

### BAL/blood eosinophil scoring (BBE score)

Using receiver operating characteristic (ROC) curve analyses, BEP was categorised using the calculated cut-off after plotting whether a patient required an additional oxygen supply. Patients were stratified into three groups by the initial PEC and BEP. Patients who underwent BAL analyses were categorised into high and low BEP groups. Patients were categorised by PEC using a cut-off of 500 × 10^9^ cells/L according to a previous study [8]. Patients with both high BEP and high PEC were given a score of 2. Patients with either a high BEP or PEC were given a score of 1. Patients with no increase in either BEP or PEC were given a score of 0.

### Statistical analyses

Statistical analyses were performed using SAS 9.3 software (SAS Institute, Cary, NC, USA). Quantitative variables are presented as means ± standard deviations, and categorical variables are shown as numbers and percentages. Categorical variables, including sex, presence of a smoking history, ICU admission history, mechanical ventilation, HFNC, bilateral lung infiltrates, pleural effusion, and use of corticosteroids were compared between the groups using a chi-square test. Continuous variables, including age, number of cigarette smoking days, WBCs, PEC/BEP, CRP, hospital days, ICU admission days, oxygen supply days, and corticosteroid treatment duration were assessed using Student’s *t*-test. We performed correlation analyses to demonstrate the correlations between BEP and the PaO_2_/FiO_2_ ratio, oxygen supply days, and PEC. We performed multiple logistic regression on significant oxygen requirement with two different models using age, C-reactive protein (CRP), smoking duration, and BBE score (model 1) and age, CRP, BEP, and PEC (model 2).

## Results

### Clinical characteristics

A total of 338 patients with AEP were evaluated. Of all, 253 (74.9%) patients underwent BAL and 221 (65.4%) patients had eosinophil percentage more than 25%. Beside these patients, 117 (34.6%) patients were diagnosed as AEP on basis of clinical and radiological context. As there was no patient who underwent lung biopsy, no patient was diagnosed by pathologic confirmation. Table 1 shows the general clinical characteristics of the patients. All but one patient was male (99.7%), and their mean age was 20.4 years. Only two patients were never smokers. Mean total number of cigarette smoking days was 26.2. Mean WBC level was 14,359. Mean PEC, BEP, and CRP levels were 4.1, 46.7%, and 9.4, respectively. About 37% of patients were initially admitted to the ICU, and mean hospital days, ICU admission days, and oxygen supply days were 10.5, 2.0, and 2.8, respectively. Three patients underwent mechanical ventilation and nine patients were oxygenated with HFNC during ICU care. The initial chest radiograph findings revealed 94.1% bilateral infiltrates and 51.2% bilateral effusion. As an initial treatment, 76.9% of patients underwent intravenous corticosteroid therapy, 18.9% underwent oral corticosteroid therapy, and 4.1% did not receive steroids. The mean duration of steroid treatment was 14.2 days.

**Table 1:** Clinical characteristics of the 338 study patients

Clinical parameters	Number
Sex (male) (n,%)	337 (99.7)
Mean age (SD)	20.4 ± 1.4
Smoking history	
Ever smoker	336 (99.4)
Never smoker	2 (0.6)
Cigarette smoking days	26.2 ± 25.4
WBC	14,359.3 ± 5562.0
Peripheral eosinophil (%)	4.1 ± 5.7
BAL eosinophil (%)	46.7 ± 17.7
CRP	9.4 ± 5.3
Hospital days	10.5 ± 4.6
ICU admission	126 (37.3)
ICU admission days (*n* = 126)	2.0 ± 1.3
Mechanical ventilation	3 (0.9)
HFNC	9 (2.7)
Oxygen supply days	2.8 ± 1.5
Chest Radiograph Findings	
Bilateral infiltrates	318 (94.1)
No effusion	122 (36.1)
Unilateral effusion	41 (12.1)
Bilateral effusion	173 (51.2)
Initial treatment	
No steroid treatment	14 (4.1)
Oral corticosteroid	64 (18.9)
Intravenous corticosteroid	260 (76.9)
Steroid treatment duration (*n* = 321)	14.2 ± 4.3

Abbreviations**:** *BAL* Bronchoalveolar lavage, *CRP* C-reactive protein, *HFNC* High-flow nasal cannula, *ICU* Intensive care unit, *SD* Standard deviation, *WBC* White blood cell

### Correlation between BEP and other significant parameters

**Fig. 1:**
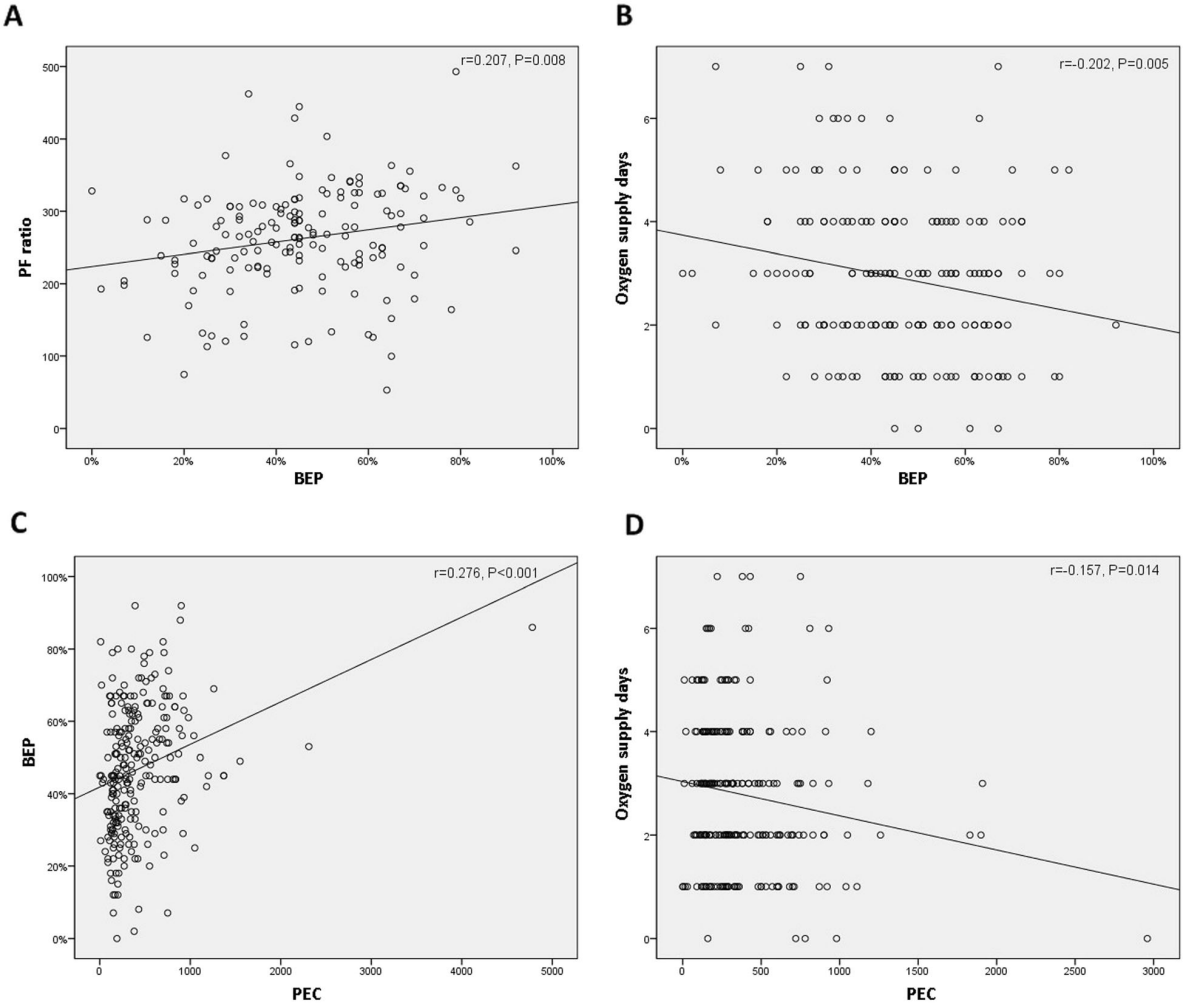
Correlation analyses between eosinophilia and significant clinical parameters; (**a**) PaO_2_/FiO_2_ and BEP; (**b**) oxygen supply days and BEP; (**c**) BEP and PEC; (**d**) number of oxygen supply days and PEC. PF ratio: PaO2/FiO2; BEP: bronchoalveolar lavage (BAL) eosinophil percentage; PEC: peripheral eosinophil count

The correlation analyses showed significant but weak correlations between the variables. BEP had a linear increasing relationship with the PaO_2_/FiO_2_ ratio (Pearson’s correlation coefficient *r* = 0.207, *P* = 0.008) (Fig. 1a). In addition, the number of oxygen supply days had a linearly decreasing relationship with BEP (Pearson’s correlation coefficient *r* = − 0.202, *P* = 0.005) (Fig. 1b). PEC had a linearly increasing relationship with BEP (Pearson’s correlation coefficient *r* = 0.276, *P* < 0.001) (Fig. 1c) and a linearly decreasing relationship with the number of oxygen supply days (Pearson’s correlation coefficient *r* = − 0.157, *P* = 0.014) (Fig. 1d).

### Comparison between groups stratified by the BBE score

**Fig. 2:**
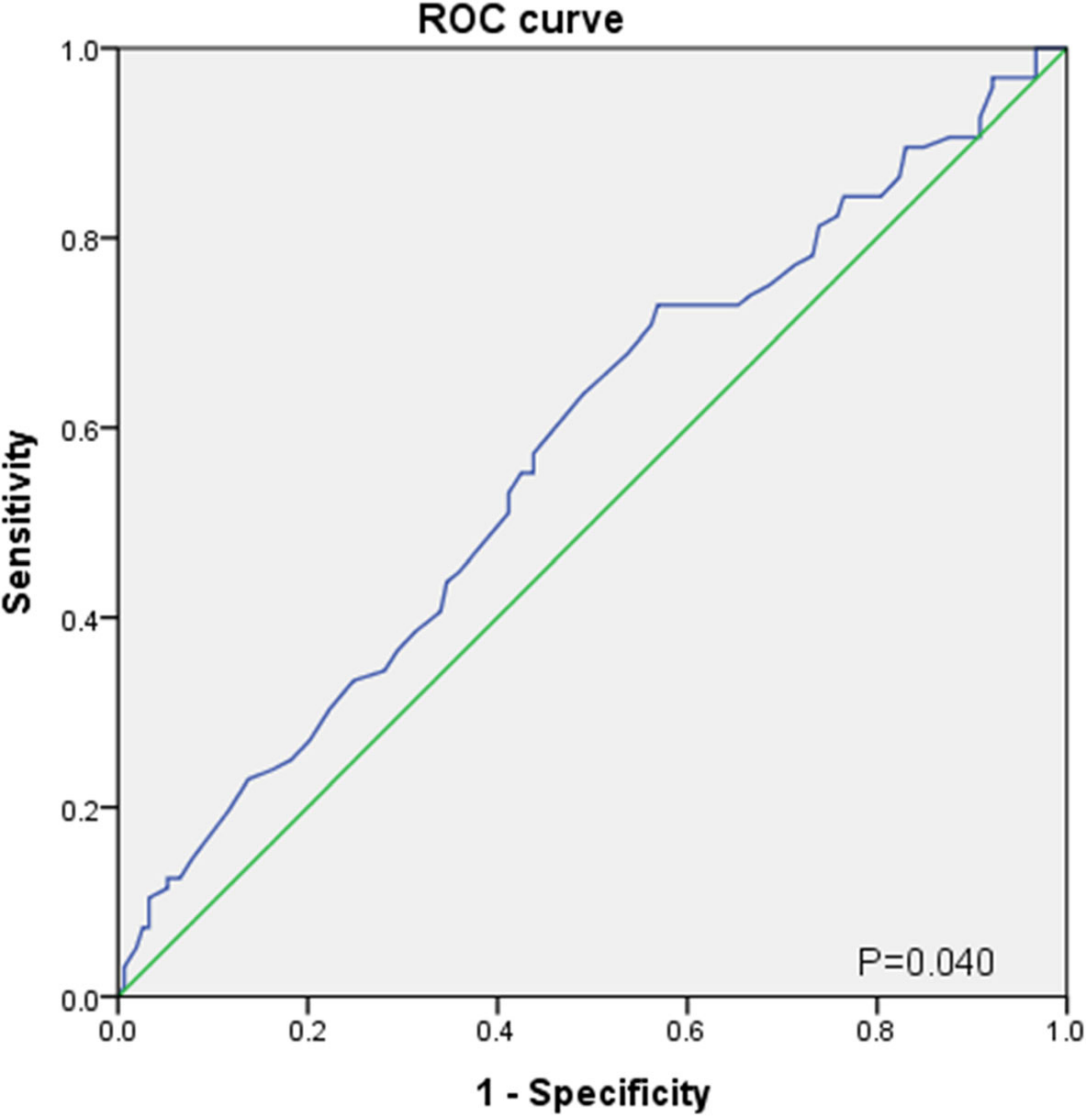
*ROC curve test for BEP as a predictor of requirement of additional oxygen supply.* AEP: acute eosinophilic pneumonia; ROC curve: Receiver operating characteristic curve

A total of 244 patients had both an initial complete blood count and BAL results for evaluation. The optimal cut-off of BEP for prediction of significant oxygen supply requirement was calculated using ROC curve analysis. (Fig. 2). The area under the ROC curve was 0.577 with a *P*-value of 0.04. The calculated cut-off was 41.5% for BEP.

The baseline clinical characteristics of the 244 patients are shown in Table 2. Mean WBC, PEC, BEP, and CRP were 14,231, 3.8, 46.8%, and 9.5, respectively. The mean number of hospital days was 11.0 and ICU admission days was 2.0. The mean PaO_2_/FiO_2_ ratio at admission was 261.9. After stratification by BBE score, 78 patients were in group with score 0, 108 patients were in group with score 1, and 58 were in group with score 2. Male proportion and mean age were not different between the groups. The proportion of ever smokers and the mean number of smoking days were not significantly different.

**Table 2:** Comparison of clinical characteristics between three AEP groups stratified by the BAL/blood eosinophil score (*n* = 244)

	Overall patients	0	1	2	*P*-value
Number of patients	244	78	108	58	–
Sex (male) (n,%)	243 (99.6)	78 (100)	108 (100)	57 (98.3)	0.200
Mean age (SD)	20.4 ± 1.5	20.3 ± 1.1	20.5 ± 1.9	20.5 ± 1.2	0.488
Smoking history					0.077
Ever smoker	243 (99.6)	78 (100)	108 (100)	57 (98.3)	
Never smoker	1 (0.4)	0 (0)	0 (0)	1 (1.7)	
Cigarette smoking days	26.9 ± 26.9	29.4 ± 34.1	24.8 ± 24.4	27.4 ± 20.3	0.545
WBC	14,231 ± 3888	16,248 ± 5005	14,489 ± 5130	11,037 ± 3888	< 0.001
Peripheral eosinophil (%)	3.8 ± 4.8	1.5 ± 1.0	2.7 ± 2.2	9.0 ± 7.1	< 0.001
Peripheral eosinophil count	419 ± 419	213 ± 100	318 ± 199	885 ± 603	< 0.001
BAL eosinophil (%)	46.8 ± 17.8	23.3 ± 9.3	53.4 ± 14.2	59.3 ± 12.4	< 0.001
BAL eosinophil count	382 ± 500	154 ± 137	502 ± 577	469 ± 558	< 0.001
CRP (unit)	9.5 ± 5.5	11.2 ± 4.9	10.2 ± 5.7	5.8 ± 3.8	< 0.001
Hospital days	11.0 ± 4.7	12.4 ± 6.3	10.6 ± 3.6	10.1 ± 3.6	0.009
ICU admission	96 (39.8)	33 (42.3)	55 (50.9)	8 (13.8)	< 0.001
ICU admission days	2.0 ± 1.3	2.1 ± 1.4	1.9 ± 1.2	2.3 ± 1.0	0.731
PF ratio at admission	261.9 ± 73.4	249.7 ± 65.8	253.8 ± 79.0	300.9 ± 59.9	0.003
O2 supply					0.031
None	52 (21.3)	11 (14.1)	20 (18.5)	21 (36.2)	
Nasal or mask only	184 (75.4)	63 (80.8)	86 (79.6)	35 (60.3)	
Mechanical ventilation	3 (1.2)	2 (2.6)	1 (0.9)	0 (0.0)	
HFNC	5 (2.0)	2 (2.6)	1 (0.9)	2 (3.4)	
Oxygen supply days	2.9 ± 1.5	3.4 ± 1.6	2.8 ± 1.4	2.3 ± 1.5	0.002
Steroid treatment days	14.3 ± 5.2	15.5 ± 5.8	14.0 ± 4.7	12.6 ± 4.7	0.006

Abbreviations**:** *BAL* Bronchoalveolar lavage, *CRP* C-reactive protein, *HFNC* High-flow nasal cannula, *ICU* Intensive care unit, *PF ratio* PaO2/FiO2, *SD* Standard deviation, *WBC* White blood cell

**Fig. 3:**
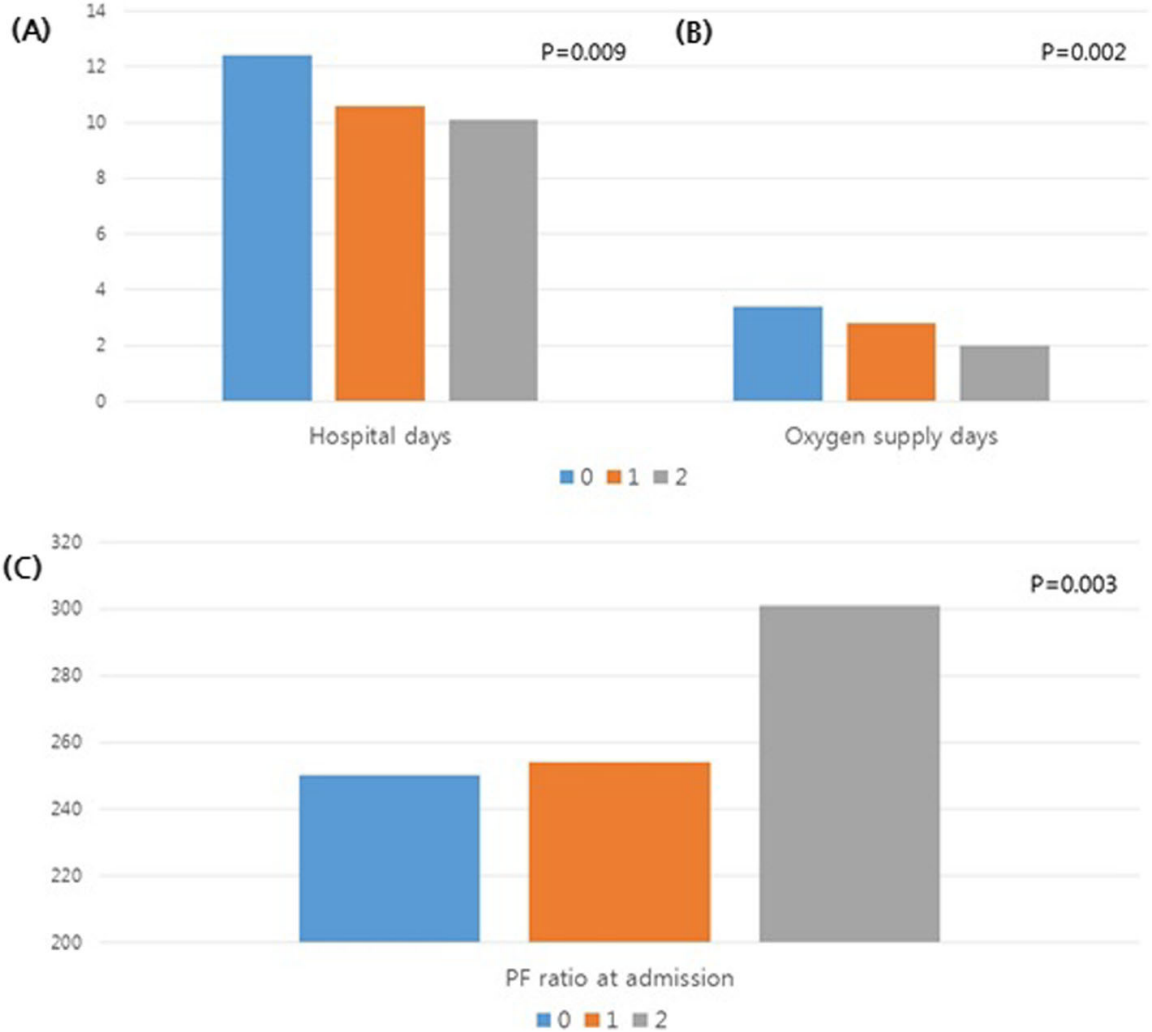
Comparison of clinical parameters between the three AEP groups stratified by the BAL/blood eosinophil score, **a**) hospital days, (**b**) oxygen supply days, and (**c**) PaO_2_/FiO_2_ at admission. AEP: acute eosinophilic pneumonia; BAL: bronchoalveolar lavage; PF ratio: PaO2/FiO2. The English in this document has been checked by at least two professional editors, both native speakers of English. For a certificate, please see: http://www.textcheck.com/certificate/XxOLy1

WBCs were significantly different between the groups. The mean WBC decreased with an increase in the BBE score. The values were 16,248, 14,489, and 11,037 for groups with score 0, 1, and 2, respectively (*P* < 0.001). Both mean PEC and BEP increased significantly as the score increased from 0 to 2 (*P* < 0.001 and *P* < 0.001, respectively). CRP was inversely related with the score (*P* < 0.001). The mean number of hospital days decreased significantly as the score increased from 0 to 2 (*P* = 0.009) (Fig. 3a). The proportion of patients who were admitted to the ICU was significantly lower in group with score 2 than in group with score 0 (*P* < 0.001). The mean duration of ICU stay was not significantly different between the groups (*P* = 0.731).

A higher proportion of patients was given additional oxygen as the score decreased from 2 to 0 (*P* = 0.031). Two patients in group with score 0 and 1 patient in group with score 1 were mechanically ventilated, but no one in group with score 2 underwent mechanical ventilation. The mean total number of days of oxygen supply was 3.4, 2.8, and 2.3 in groups with score 0, 1, and 2, respectively (*P* = 0.002) (Fig. 3b), while the mean number of steroid treatment days was 15.5, 14.0, and 12.6, respectively (*P* = 0.006). The initial PaO_2_/FiO_2_ ratios were 249.7, 253.8, and 300.9, respectively (*P* = 0.003) (Fig. 3c).

### Association with significant oxygen requirements

Age, CRP, smoking duration before admission, BEP, PEC, and BBE score were entered into logistic regression analyses to determine the association with significant oxygen requirements (Table 3). CRP, BEP (categorical), PEC, and BBE scoring were significant in univariate analyses. Two models were applied for multivariate analyses. In model 1, BBE scoring was entered, while BEP and PEC were excluded. In model 2, BEP and PEC were included, while BBE score was excluded. In model 1, BBE score was the only significant factor associated with significant oxygen requirements (*P* = 0.018). Compared to group with score 2, the odds ratio (OR) for significant oxygen requirements was 2.25 (95% confidence interval [CI]. 1.112–4.574, *P* = 0.024) for group with score 1, while the OR was 3.014 (95% CI 1.378–6.593, *P* = 0.006) for group with score 2. In model 2, BEP was a significant factor (OR 1.939, 95% CI 1.072–3.505, *P* = 0.028).

**Table 3:** Patient factors analysis showing association with significant oxygen requirements

	Univariate			Multiple	(Model 1)		Multiple	(Model 2)	
Characteristics	OR	95% CI	P	OR	95% CI	P	OR	95% CI	P
Age^a^	1.140	0.969–1.363	0.13	1.229	0.972–1.553	0.084	1.226	0.974–1.543	0.083
CRP	1.070	1.020–1.110	0.009	1.022	0.968–1.078	0.432	1.026	0.972–1.083	0.356
Smoking duration	1.006	0.997–1.016	0.201	–	–	–	–	–	–
BAL eosinophil % (High/low)	0.490	0.282–0.851	0.011				0.516	0.285–0.933	0.028
Peripheral eosinophil count (High/low)	0.497	0.283–0.873	0.015				0.679	0.358–1.286	0.234
Blood/BAL scoring									
0	3.187	(1.567–6.484)	0.001	3.014	1.378–6.593	0.006			
1	2.610	(1.356–5.024)	0.004	2.256	1.112–4.574	0.024			
2	1	–	0.003	1	–	0.018			

^a^For every 1 year increase

Model 1: Blood/BAL scoring is entered for multivariate analysis

Model 2: BAL eosinophil (%) (High/low) and blood eosinophil count were entered for multivariate analysis. Blood/BAL scoring were excluded from analysis

Abbreviations: *BAL* Bronchoalveolar lavage, *CRP* C-reactive protein

## Discussion

The disease severity and clinical course of AEP vary in mild to severe cases, which may require no oxygen supply for mechanical ventilation. Therefore, it may be important to assess patients who are suspected of having AEP at initial presentation. However, it is difficult to reveal the prognostic factors of the disease statistically because it is a relatively rare disease. As patients from all military hospitals who are suspected of having AEP in South Korea are referred to the AFCH, we were able to collect nationwide military data, although it was a single centre study. The development of AEP is associated with newly started or a recent resumption of cigarette smoking [17, 18]. Many soldiers start or resume cigarette smoking, especially conscripted soldiers, as many have only recently attained the minimum age to consume cigarettes legally. Thus, we were able to enrol a sizable number of patients in this study.

We investigated factors that may affect disease severity and the clinical course of patients with AEP. Despite no statistical significance in multivariate analyses, we found an association between peripheral eosinophilia and decreased oxygen requirements in univariate analyses. Furthermore, PEC was negatively correlated with the number of oxygen supply days. Previous studies have reported that patients with initial peripheral eosinophilia undergo a mild disease course compared to patients with normal levels of eosinophils [12, 13].

The exact mechanism of this association has not been revealed; however, it may be associated with cytokines, such as interleukin (IL)-5, which have an inverse correlation with PEC [19]. As IL-5 is an important mediator of recruitment of eosinophils from peripheral blood into the lungs, a decrease in IL-5 may attenuate eosinophilic inflammation in the lung. In addition, PEC tends to increase during the course of AEP [3, 20–24]. This suggests that patients with initial peripheral eosinophilia may present to the medical centre at a later course of the disease than patients with a normal PEC. As the prognosis of AEP was favourable in this subgroup of our patients, we assumed that patients with initial peripheral eosinophilia already passed the peak of disease activity before admission and were admitted at a later course of the disease.

BEP may also predict clinical outcomes. Our results are in line with a previous study that showed that patients with peripheral eosinophilia have a significantly higher BEP [12]. Furthermore, Sine et al. revealed a positive correlation between BAL eosinophilia and the PaO_2_/FiO_2_ ratio in patients with AEP [13]. In our study, we found that BEP was positively correlated with PaO_2_/FiO_2_ but negatively correlated with total oxygen supply days, suggesting that patients with higher BEP may experience a milder clinical course. Moreover, an increase in BEP was independently associated with decreased oxygen requirements. However, the correlation coefficients were weak to suggest strong correlations, which should be taken into account.

Using PEC alone, a previous study was unable to predict important outcomes, such as the number of hospital days or the PaO2/FiO2 ratio [10]. In our study, we also evaluated the association between the combination of the two parameters (BEP an PEC) and clinical outcomes. After stratifying by BBE score, group with score 2 (elevated BEP and PEC) had a lower CRP, hospital days, ICU admission, PaO_2_/FiO_2_ ratio, total oxygen supply days, and steroid treatment days compared to the other groups. Multiple logistic regression models revealed that BBE score was the only factor that predicted a lower chance of a significant oxygen requirement. Nevertheless, the application of BBE scoring to other AEP population needs further validation process, because the cut-off used to categorize BEP into high/low group could be different in other patients group. However, our findings suggest that elevated BEP and PEC result in a more favourable clinical outcome in patients with AEP.

Some limitations of our study should be discussed. First, as we used retrospective data, the possibility of selection bias or observer bias should be considered. Second, we defined “significant oxygen requirement” as the total number of days of oxygen supplied equal to 2 days or more during admission. Although this definition was made after considering the possibility of an oxygen supplement related to bronchoscopy, whether this definition is appropriate should be validated in future studies. Third, patients with a milder clinical course may not have presented to our hospital. AEP with mild symptoms and little or no hypoxemia may resolve without treatment. Therefore, some patients with a mild clinical course may have been neglected as they may have not presented to our centre. Fourth, the AUC obtained from ROC curve analysis, while determining the optimal cutoff value of BEP for prediction of significant oxygen requirement was relatively low (AUC = 0.577). Finally, as our study was performed in a military hospital, the enrolled patients were mostly young males. Thus, our study population may not be representative of the general population. However, as smoking-related AEP is likely to develop soon after patients start to smoke, our results retain clinical importance.

## Conclusions

In conclusion, AEP patients with both peripheral and BAL eosinophilia at presentation have more favourable outcomes. Prospective studies that apply this scoring system to different AEP populations are necessary for further clinical application.

## Data Availability

The database used in this study are available from the corresponding author on reasonable request.

## Abbreviations

AEP: Acute eosinophilic pneumonia
AFCH: The army forces capital hospital
BAL: Bronchoalveolar lavage
BBE score: BAL/blood eosinophil score
BEP: Bronchoalveolar lavage eosinophil percentage
CRP: C-reactive protein
EMR: Electronic medical records
FiO2: Inspiratory oxygen fraction
HFNC: High-flow nasal cannula
ICU: Intensive care unit
IL-5: Interleukin-5
PACS: Picture archiving and communication system
PaO2: Arterial oxygen tension
PEC: Peripheral eosinophil count.
ROC: Curve receiver operating characteristic curve.
WBC: White blood cell.
